# Social monitoring in a multilevel society: a playback study with male Guinea baboons

**DOI:** 10.1007/s00265-012-1425-1

**Published:** 2012-10-11

**Authors:** Peter Maciej, Annika Patzelt, Ibrahima Ndao, Kurt Hammerschmidt, Julia Fischer

**Affiliations:** 1 Cognitive Ethology Laboratory, German Primate Center, Kellnerweg 4, 37077 Göttingen, Germany; 2 Courant Research Centre “Evolution of Social Behaviour”, 37077 Göttingen, Germany; 3 Direction de Park National de Niokolo-Koba, Tambacounda, Senegal

**Keywords:** Guinea baboon, Multilevel societies, *Papio papio*, Social attention, Social interactions

## Abstract

Keeping track of social interactions among conspecifics is a driving force for the evolution of social cognition. How social cognition, such as social knowledge, ties in with a species' social organization is, however, largely unexplored. We investigated the social knowledge of wild Guinea baboons (*Papio papio*) ranging in Senegal, a species that lives in a fluid multilevel society with overlapping habitat use. Using playback experiments, we tested how adult males differentiate between subjects from their own vs. a neighboring or a stranger social unit (“gang”) and assessed ranging patterns with Global Positioning System (GPS) data. While territorial species usually differentiate between group and nongroup members and often respond more strongly to strangers than neighbors (the “dear enemy” effect), subjects in this highly tolerant species should largely ignore other unit members and mainly attend to subjects from their own unit. Males responded strongly after playback of calls recorded from members of their own gang, while they attended only briefly to neighbor or stranger calls. Apparently, males benefit from monitoring the social maneuvers in their own social unit, while it remains to be resolved whether they are unmotivated or unable to keep track of the identities and actions of individuals outside their own gang. The study highlights how the allocation of social attention is tuned to the specifics of a species' social organization, while a complex social organization does not necessarily translate into the need for more elaborate social knowledge.

## Introduction

Social living is widespread across animal taxa, but there is considerable variation in terms of the frequency, nature, and consistency of social interactions among group members. According to the social complexity hypothesis, keeping track of the social interactions within one's group is a major driving force for brain evolution and intelligence (Dunbar and Shultz 2007; Dunbar 2011; Freeberg et al. 2012). One constitutive element of social cognition is social knowledge, that is, the recognition of individuals and their classification according to different social attributes, including their relationships with third parties (Cheney and Seyfarth 2007; Fischer 2012). Yet, little is known how social knowledge is adapted to, or influenced by, a species' social organization. Recently, it was hypothesized that life in groups with fission–fusion dynamics may add an additional layer of social complexity, as animals do not only have to keep track of the interactions in their own group but also process the composition of other subgroups and negotiate the merging and splitting of units (Amici et al. 2008; Aureli et al. 2008).

To date, social knowledge has been investigated at different levels, including individual recognition, categorization according to social attributes, and group membership recognition. Regarding individual recognition, there are numerous studies showing that kin recognize each other vocally (Rendall et al. 1996; Hammerschmidt and Fischer 1998; Charrier et al. 2003; Fischer 2004; Müller and Manser 2008). There is also evidence that nonhuman primates living in stable groups are able to classify other individuals with regard to different social attributes, such as dominance rank or bond strength (Soltis et al. 2002; Bergman et al. 2003; Kitchen et al. 2005; Range 2005). In addition, animals living in stable groups have been shown to distinguish between members of their own group and those belonging to other groups (e.g., Cheney and Seyfarth 1982; Hopp et al. 2001; Wich et al. 2002; Range 2005; Meunier et al. 2012 for the vocal domain; Schell et al. 2011 for the visual domain). Importantly, in which way subjects respond to neighbors or strangers may not only depend on the competitive regime between groups but also on the status of the individuals. Males typically consider other males as competitors, while lactating females may fear infanticidal attacks (Steenbeek 1999; van Schaik and Janson 2000; Ren et al. 2011). In contrast, cycling females may be attracted to neighboring or stranger males (Cheney and Seyfarth 1987; Palombit 1994; Agoramoorthy and Hsu 2000).

In territorial species, individuals may distinguish among different neighboring groups (Cheney and Seyfarth 1982; Hyman and Hughes 2006) and differentiate between members of stranger vs. adjacent groups. According to Fisher, territory owners may respond less strongly to neighboring group members because subjects are familiar with each other (the so-called “dear enemy” effect; Fisher 1954), and there is ample evidence for this phenomenon (e.g., Temeles 1994; Wich et al. 2002; Radford 2005). In contrast, when territory boundaries are at stake, the reverse “nasty neighbour” effect may set in (Temeles 1990; Müller and Manser 2007; Herbinger et al. 2009).

Not all socially living species live in clearly delineated social units with well-defined intergroup boundaries but may exhibit a rather fluid social organization instead. Such complex multilevel social systems are characterized by low territoriality and frequently changing animal aggregations (e.g., hamadryas baboons (*Papio hamdryas*) (Kummer 1968); gelada baboons (*Theropithecus gelada*) (Snyder-Mackler et al. 2012); snub-nosed monkeys (*Rhinopithecus bieti*) (Ren et al. 2012)). In such cases, the responses to neighbors or strangers may be less pronounced.

In the present study, we focused on Guinea baboons and tested neighbor–stranger discrimination in males. Specifically, we assessed how males differentiate between subjects from their own social unit, a neighboring social unit, and stranger males. The broader aim is to understand the relationship between social organization and social knowledge using baboons as a model. Guinea baboons exhibit a complex multilevel social organization that differs substantially from the stable groups reported for savannah baboons (chacma, olive, and yellow baboons), the male-centered harem structure found in hamadryas baboons, or the female-centered one-male units in gelada baboons (Galat-Luong et al. 2006; Patzelt et al. 2011; Swedell 2011).

Guinea baboons live in multimale, multifemale units, comprising 50–70 individuals, which we termed “gangs,” to differentiate clearly between the multilevel social organization of this species and the stable social groups of savannah baboons. Neighboring gangs aggregate frequently during the day and at sleeping sites and can form large communities of more than 350 individuals (Dunbar and Nathan 1972; Galat-Luong et al. 2006; Patzelt et al. 2011). Despite the extensive spatial overlap in territory use by different gangs and the frequent close proximity of individuals belonging to different gangs, sociopositive as well as socionegative interactions are largely restricted to members of the own gang (AP, PM, IN, D Zinner, JF, unpublished data).

To investigate how adult male Guinea baboons differentiate between their own, neighboring, and stranger gang males, we conducted playback experiments. We also assessed the spatial and temporal overlaps between gangs using Global Positioning System (GPS) data from collared individuals. In the playback experiments, we broadcasted male grunts from different categories (“own gang,” “neighboring gang,” and “stranger gang”). According to the theory outlined above, we would expect that Guinea baboon males respond strongly to stranger, but not to neighboring males, as they do not consider these males as potential threats (the “dear enemy” effect). Alternatively, because of the high tolerance, they may not consider any members from other gangs as competitors and thus, largely ignore them. In this case, it might be that they focus their social attention on the members of their own gang and thus, respond most strongly after presentation of calls from their own gang members.

## Methods

### Study site

Research took place from March 2011 until July 2011 at the field station of the German Primate Center (DPZ), the Centre de Recherche de Primatologie (CRP), in the Simenti region (13°01′34″N, 13°17′41″W). The area is located in the Niokolo-Koba National Park in southeastern Senegal. The climate in this region is highly seasonal with a dry season from November until June and a rainy season from July until October. The annual rainfall ranges from 1,000 to 1,100 mm (Dupuy 1971) and occurs mainyl in the rainy season. The vegetation in this area varies from grassland savannah to deciduous forest, with gallery forest limited to the river banks. Despite a dramatic decrease in large mammal population sizes during the last decades, potential predators such as lions (*Panthera leo*), leopards (*Panthera pardus*), and spotted hyenas (*Crocuta crocuta*) still exist in this region (Ndao and Henschel 2011).

### Subjects

**Fig. 1:**
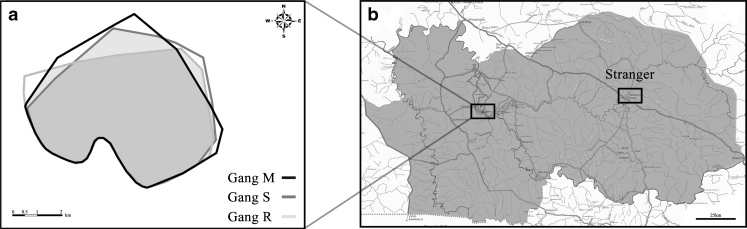
Recording sites of male grunt vocalizations. In **a**, the home ranges of three neighboring gangs of the study area were calculated by using GPS points of six collared individuals (*M* Mare gang, *S* Simenti gang, *R* River gang). **b** A simplified map of the Niokolo-Koba National Park and the recording site of stranger males' vocalizations

The Guinea baboon community within the research area comprises approximately 350–400 individuals, separated into several gangs. Twelve subjects were fitted with radio and GPS collars (Tellus GPS, Televilt, Sweden). Systematic observation began in 2010 and since June 2010, two gangs are fully and one gang is semihabituated to humans (Mare gang = M, Simenti gang = S, River gang = R, i.e., around 200 individuals), which makes detailed focal observations possible. GPS fixes from six individuals in the three gangs were used (two collars per gang) to analyze spatiotemporal interactions between gangs. GPS fixes during the day were taken every 2 h and during the night every 3 h. By using a Ultra High Frequency (UHF) download system (RCD-04, Televilt, Sweden), GPS fixes were downloaded from the collars. We analyzed GPS data for 5 months during the dry season (*X* = 1,970 GPS points per individual). We calculated home ranges of the three gangs via minimum convex polygons (MCP) (ArcGIS 2010, Esri, Inc., Redlands, US, the MCP of the three neighboring gangs are illustrated in Fig. 1a). The software “at” (programmed by Ch. Franzl) was used to assess the spatial distance between the three gangs by calculating, from all possible GPS points in the given period, the percentage of points the gangs spend within a radius of 500 m of each other (*X* = 25.52 ± 3.37 %).

To date, the dispersal pattern of Guinea baboons remains unclear, although preliminary observations suggest that females transfer between gangs (A Goffe, G Fickenscher, D Zinner, JF, unpublished data). Competition for access to females between males appears to be rare and altogether clearly different from male competition witnessed in chacma baboons (U Kalbitzer, JF, personal observations). Males maintain strong bonds with specific other males and engage in both grooming and ritualized greetings (Whitham and Maestripieri 2003). Because of the scarcity of agonistic interactions among Guinea baboon males within a gang, it was not possible to establish a dominance hierarchy for the animals tested in this study.

Gang aggregations of Guinea baboons are flexible and may vary from day to day (AP, PM, IN, D Zinner, JF, unpublished data). During aggregations, members of different gangs may come in close proximity to one another, so that it is difficult to delineate one gang from another (Galat-Luong et al. 2006; Patzelt et al. 2011).

### Experimental design and data analysis

**Fig. 2:**
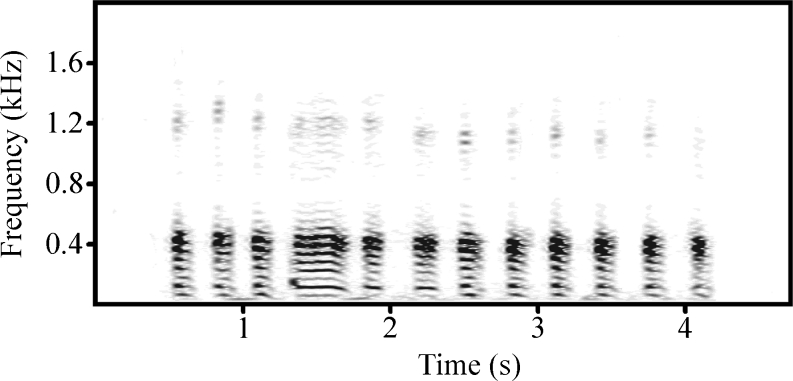
A playback sequence of a male grunt bout. The spectrogram was created by using Avisoft-SASLab Pro 5.1 (R. Specht, Berlin, Germany; fast Fourier transform resolution 1.024 points, sampling frequency: 4 kHz, time resolution: 4 ms, time overlap: 98.43, Hamming window)

We simulated the presence of another male by playing back a bout of male grunts. Grunt vocalizations are the most common vocalizations in baboons and are individually distinctive (Owren et al. 1997; Rendall 2003). In other baboon species (Rendall et al. 1999; Meise et al. 2011), they are typically given as a signal of “benign intent” (Cheney and Seyfarth 1997) when one animal approaches another to engage in sociopositive behavior. They may also function as “contact calls” when animals are initiating a move (Fischer and Zinner 2011) or while traveling (Rendall et al. 1999; Meise et al. 2011). For all experiments, we used naturally occurring sequences of eight to 12 grunts which were matched for duration (≈4 s); Fig. 2 shows a spectrogram of a male grunt bout. The amplitude of the call sequences was adjusted by using a Voltcraft 322 sound level meter (Voltcraft, Germany; ‘C’ weighting, response time: 125 ms) and sound pressure level was kept constant (*X* = 70.4 ± 1.8 dB, measured at 1.5 m distance from the loudspeaker). Call sequences were recorded during focal sampling from March 2010 until June 2010 and February 2011 until March 2011, using a Marantz solid state recorder PMD 661 and a Sennheiser ME 66 directional microphone. Only calls recorded from less than 6 m and with a high signal-to-noise ratio were used for the experiments. Calls from stranger males were recorded from a baboon community ranging more than 60 km away from our study area, as depicted in Fig. 1b.

Grunts were played back to eight males from gang M and four males from gang S in a balanced order. In total, we conducted 36 playback trials with the 12 males (11 were adult, one was just reaching adulthood). Calls of both neighboring gangs of gang M and gang S were used in the neighbor condition. Each male was tested no more than once per week to minimize habituation effects. As members of the same gang were rarely far away from each other, playback trials were only conducted when the caller had been seen in the gang that day, but had been out of sight for at least 15 min. In all trials, the loudspeaker was hidden behind large objects (e.g., termite hills and tree trunks), 10–15 m away from the caller. Calls were played back using an active speaker (David Active, VISONIK, Berlin) connected to a Marantz PMD-661 recorder. Although the ability to conduct the playbacks was limited by natural conditions (presence of the caller and dense vegetation), we attempted to playback calls only in situations when the subject was at the edge of the gang and did not socially interact or was in physical contact with any other individual. In two trials, however, another individual came within a distance of 10 m to the subject during the experiment but did not interact socially with it. Altogether, each male was tested a total of three times.

We filmed all playback experiments using a Sony Handycam (DCR-HC90E). Subsequently, we digitized the videotapes and saved them in Windows Media video format. We did a frame-by-frame analysis using VirtualDub 1.9.11 (freeware, www.virtualdub.org). We examined the subjects' reaction by scoring the orienting response in one of the four categories: 1: no reaction, 2: looking in the direction of the speaker, 3: approaching (walking toward the speaker, which may include looking towards the speaker), and 4: approaching the speaker and vocalizing while approaching. We further determined the duration of the orienting response within 30 s following the onset of the playback. In cases of several orienting responses within 30 s, we summed the duration of each orienting response (irrespective of the response category). Finally, we investigated the latency to respond (the time between the onset of the call and the onset of the orienting response). A subset of randomly selected trials was “blind”-coded by a second observer, who was unaware of the experimental conditions. Cronbachs' alpha for response duration and response latency was 0.988 (*n* = 18) and 0.975 (*n* = 18), indicating excellent interobserver reliability. For the behavioral categories, the interobserver reliability was similarly high (Cronbachs' alpha = 0.984 (*n* = 18)). We used exact Friedman tests and exact Wilcoxon signed-rank tests to analyze the differences in relation to category, and exact Mann–Whitney *U* test to consider possible differences between the two gangs (Mundry and Fischer 1998). All tests were two-tailed and significance level was set at 0.05. SPSS 19 was used for the analysis.

## Results

Subjects oriented toward the speaker in 35 out of 36 trials. There were no significant differences in the responses between the two gangs in any of the conditions (Mann–Whitney *U* test, own gang: *n*_*M*_ = 8, *n*_*S*_ = 4, *W* = 22.5, *p* = 0.384; neighbor gang: *W* = 20, *p* = 0.333; stranger gang: *W* = 20, *p* = 0.333). Subsequently, we pooled the data of both gangs and analyzed the differences in relation to the call categories. The behavioral scores (no reaction, looking, approaching, and approaching plus vocalizing) differed significantly between the different playback conditions (Friedman test, *k* = 3, *n* = 12 *χ* ^2^ = 14, *p* = 0.001). Males responded most strongly to their own gang members' calls compared to neighboring and stranger males' calls. Post hoc comparison revealed a significant difference between nongang members' calls: own vs. neighboring (Wilcoxon signed-rank test, *n* = 12, *W* = 28, *p* = 0.016) and own vs. stranger gangs (Wilcoxon signed-rank test, *n* = 12, *W* = 28, *p* = 0.016), but no difference was found in the response behavior between neighboring and stranger males' calls (Wilcoxon signed-rank test, *n* = 12, *W* = 0, *p* = 1). The results of the post hoc tests still remained significant after Bonferroni correction (*p* = 0.017). In seven out of the 12 trials, the male approached the loudspeaker when calls from the same gang were played back. Grunt vocalization occurred only once, in response to the calls from the same gang. Neither approaches nor vocalizations occurred in response to calls of member from other gangs.

**Fig. 3:**
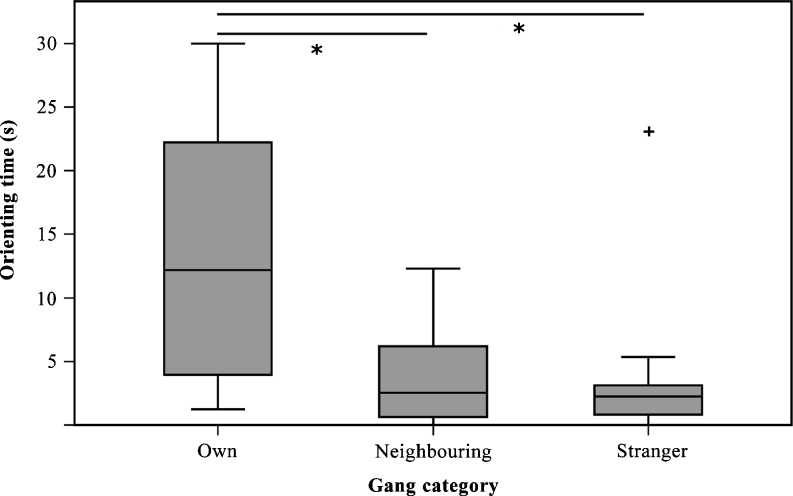
Orienting time towards the speaker. Median is shown as a *black line*. *Box plots* represent quartiles and *error bars* represent 95 % confidence interval. *Asterisk* statistical significance, *plus sign* outliers

The observed pattern of the response duration corroborates the previous findings. Differences between the two gangs were not significant for all conditions (Mann–Whitney *U* test, own gang: *n*_*M*_ = 8, *n*_*S*_ = 4, *W* = 18, *p* = 0.808; neighbor gang: *W* = 23.5, *p* = 0.230; stranger gang: *W* = 18, *p* = 0.808). There was a significant difference across the three playback conditions (Friedman test, *n* = 12, *χ* ^2^ = 10.34, *p* = 0.004). In response to calls from the same gang, the orienting time was significantly longer (*X* = 14.3 ± 12.55 s) compared to the orienting time towards calls from neighboring gangs (*X* = 4.02 ± 3.16 s, Wilcoxon signed-rank test, *n* = 12, *W* = 69.5, *p* = 0.013) and stranger gangs (*X* = 1.95 ± 1.59 s, Wilcoxon signed-rank test, *n* = 12, *W* = 71, *p* = 0.009), as shown in Fig. 3. We found no differences in the orienting time between neighboring and stranger gang vocalizations (Wilcoxon signed-rank test, *n* = 12, *W* = 40, *p* = 0.577). The response latency did not differ across the different playback conditions (Friedman test, *n* = 12, *χ* ^2^ = 0.326, *p* = 0.906).

## Discussion

In the playback experiments, male Guinea baboons showed a significantly longer orienting behavior towards the loudspeaker after being presented with vocalizations of their own gang members than in the other two conditions; in seven out of 12 trials, they even interrupted their previous activity and approached the loudspeaker. In contrast, they only looked briefly in response to calls of males that did not belong to their own gang. There was no difference in the orienting time following the playback of neighbor or stranger males' calls. These findings are in line with the view that male Guinea baboons do not consider males from other gangs as intruders or direct competitors, as they showed a remarkably unconcerned response toward members from other gangs and focus their social attention mainly on members of their own gang.

Generally, the response pattern indicates that Guinea baboon males discriminate the vocal characteristics of their gang members and differentiate between gang and nongang members. Within their own gang, Guinea baboons are permanently associated and frequently engage in sociopositive as well as socionegative interactions with other gang members (AP, PM, IN, D Zinner, JF, unpublished data). Hence, the own gang members differ in their social relevance compared to neighboring individuals with whom gang members show only spatial proximity. The distinction depending on the consistency of social interactions and the ability to differentiate between group (or unit) vs. nongroup (or nonunit) members has also been found in other fluid animals societies, although the specific response pattern may differ (African elephants (*Loxodonta africana*) (McComb et al. 2000); sooty mangabeys (*Cercocebus torquatus atys*) (Range 2005); spider monkeys (*Ateles geoffroyi*) (Ramos-Fernandez 2005), and ravens (*Corvus corax*) (Boeckle and Bugnyar 2012)). African elephants, for example, live in large multilevel societies with a high fission–fusion dynamic (McComb et al. 2000). Although neighboring units regularly aggregate and largely overlap, strong social associations only exist between members of their own family units or bond groups. A previous study has shown that individuals distinguish between calls of their unit members and nonunit members and, while subjects engaged in a high amount of contact calling and affiliative approaches toward their unit members' calls, they largely ignore vocalizations of neighboring nonunit individuals (McComb et al. 2000). Unit members mingle frequently around nonunit individuals and are often visually separated from each other, hence to stay in contact with their social allies, via contact calling, and to discriminate their associates' calls seems to be essential in the fluid multilevel society of African elephants.

Another playback study conducted on female sooty mangabeys showed a similar discrimination pattern, albeit with a different response pattern (Range 2005). In sooty mangabeys, different sexes employ different group membership strategies. While males may join and leave the group for months, females remain resident all year round (Range et al. 2007). Neighboring nongroup males are encountered frequently and sometimes walk through the group for several minutes; however, they only rarely interact with females and are usually viewed as infanticidal threat. Resident males, in contrast, associate and interact regularly with their group members. Playback experiments have shown that females recognize the residence status of the males, irrespective of whether males are full- or part-time residents, while they respond strongly only to nonresident males, i.e., they often leave their position when calls from nonresident males were played back. Thus, in different species, the distinction of own group (or unit) vs. neighboring group (or unit) members may be driven by different selective pressures.

The long orienting time and approach responses toward their own gang members' vocalizations suggest that Guinea baboon males are attentive to the social interactions of members of their own gang. Although we are unable to determine how social attributes (such as kinship) influence the response behavior in Guinea baboons, our findings lend support to the notion that the gang constitutes the social entity within the multilevel Guinea baboon society. In other baboon taxa, such as chacma baboons, males exhibit enduring relationships with other group members, such as ‘friendships’ with lactating females (Nguyen et al. 2009; Huchard et al. 2010), consortships with oestrous females (Crockford et al. 2007) and dominance relationships with adult males (Kitchen et al. 2003). Previous playback studies have demonstrated that male and female subjects pay particular attention to the calls of their associates and are sensitive to the social events their bond partners and/or competitors are involved in (Bergman et al. 2006; Crockford et al. 2007; Lemasson et al. 2008). Rank differences, at least, seem not to explain the response pattern in Guinea baboons, since this concept appears not to be suited to characterize Guinea baboon male relationships and neither does the strength of the relationship predict response strength (AP, PM, IN, D Zinner, JF, unpublished data). Further observations and field experiments will be needed to clarify by which social attributes Guinea baboons differentiate among their gang members and how well individuals of different sexes and age classes track their social relationships.

In contrast to gang member and nongang member distinction, Guinea baboon males do not differ in their response behavior toward neighboring and stranger males and largely ignore any nongang member, irrespective of familiarity; that is, they neither show a “dear enemy” nor “nasty neighbour” effect. While at present, we are unable to clarify whether this is due to an inability to distinguish between such a large number of voices or a lack of motivation to do so, such lack of concern about potential unfamiliar individuals lies in sharp contrast to other baboon species and many group-living primates (Wich et al. 2002; Kitchen et al. 2004; Herbinger et al. 2009; Meunier et al. 2012). Theory predicts that neighbor–stranger differentiation should mostly occur when intergroup competition is high (e.g., Radford 2005; Müller and Manser 2007; Herbinger et al. 2009). Guinea baboon males do not seem to consider members from other gangs as competitors and by ignoring any nongang members' call, they justify their classification as a highly tolerant species. A playback study conducted on gelada baboon males revealed a similar lack of responses to the playback of males outside the harem holders' social unit (Bergman 2010). The author pointed out that through the absence of competitive relationships between gelada baboon harem holders, they do not need to differentiate among their unrelated neighboring units who they regularly encounter.

Another possibility might be that Guinea baboon males are simply unable to recognize all of their neighbors' call characteristics. A Guinea baboon community can comprise more than 350 individuals and the composition of subgroups varies substantially over time (Galat-Luong et al. 2006; Patzelt et al. 2011). Savannah baboons, such as chacma or olive baboons, in contrast, live in stable, medium-sized social groups with individual numbers ranging from 20 to 80 animals (Swedell 2011). Possibly, the recognition and discrimination of all males in the vicinity may be beyond the limits of Guinea baboon males. From our results, we are unable to decide whether a lack of ability or a lack of motivation accounts for the observed response pattern. Yet, the experiments clearly demonstrate that Guinea baboon males are able to discriminate the voice characteristics of their own gang members from those not belonging to their own social unit.

Our findings have important implications for the assumption that social complexity is a driving force in the evolution of social cognition. The costs and benefits, that is, the fitness consequences of tracking social interactions between others appears to be more important than the multilayered structure of the society per se. It may indeed be likely that high levels of competition or direct links between stable social bonds and reproductive success (Silk et al. 2003; Silk et al. 2009) are more decisive for the evolution of a “Machiavellian” mind (Byrne and Whiten 1988) than fission–fusion dynamics. In sum, our playback experiments showed that males took interest only in the (simulated) social interactions of their own gang members, while they ignored both neighbor and stranger calls. The generally low responsiveness toward males from other gangs is probably an expression of the low level of competition between gangs. Our findings suggest that Guinea baboon males' social relationships are characterized by the quality and consistency of the social interactions, while simple spatial aggregation does not appear to be important. Taken together, our study supports the view that the animals allocate their social attention in relation to the requirements and selective pressures generated by the specific social system they live in, while a complex social organization does not necessarily translate into the need for more elaborate social knowledge.
